# Microbial biotechnology and beyond: A roadmap for sustainable development and climate mitigation in the transition from fossil fuels to green chemistry

**DOI:** 10.1111/1751-7915.14434

**Published:** 2024-03-11

**Authors:** Juan‐Luis Ramos, Ana Segura

**Affiliations:** ^1^ Consejo Superior de Investigaciones Científicas Estación Experimental del Zaidín Granada Spain

## Abstract

Our planet, which operates as a closed system, is facing increasing entropy due to human activities such as the overexploitation of natural resources and fossil fuel use. The COP28 in Dubai emphasized the urgency to abandon fossil fuels, recognizing them as the primary cause of human‐induced environmental changes, while highlighting the need to transition to renewable energies. We promote the crucial role of microbes for sustaining biogenic cycles to combat climate change and the economic potential of synthetic biology tools for producing diverse non‐fossil fuels and chemicals, thus contributing to emission reduction in transport and industry. The shift to ‘green chemistry’ encounters challenges, derived from the availability of non‐food residues and waste (mainly lignocellulosic) as raw material, the construction of cost‐effective bioprocessing plants, product recovery from fermentation broths and the utilization of leftover lignin residues for synthesizing new chemicals, aligning with circular economy and sustainable development goals. To meet the Paris Agreement goals, an urgent global shift to low‐carbon, renewable sources is imperative, ultimately leading to the cessation of our reliance on fossil fuels.

The Earth operates as a closed system, with limited matter exchange with the surrounding universe. Human activities, such as natural resource consumption and waste generation, have heightened entropy (Ripple et al., 2023). The United Nations Climate Change Conference, COP28, held in Dubai, underscored the undeniable impact of anthropogenic contamination and fossil–fuel use, evident in, global challenges such as rising temperatures, severe droughts, wildfires and widespread floods (Maximillian et al., 2019; Ripple et al., 2023; UNCC, 2023), without forgetting biodiversity loss, ocean acidification, zoonosis and others, all linked to human behaviour (Merz et al., 2023). Merz et al. (2023) criticize that current interventions are resource‐intensive and often progress at a slow pace, which frequently treats the symptoms rather than addressing the root of maladaptive behaviour.

The growing global population and our materialistic lifestyles have exacerbated energy consumption and pollution. Fossil fuels have been pivotal for progress, but come with environmental consequences that are significant contributors to ecological issues (IPCC, 2014, 2019; UNCC, 2023). The estimated timeframe to achieve the challenge of limiting temperature increases to 1.5°C above pre‐industrial records is projected to be from 2030 to 2050 (Betts et al., 2023; Lazarus & Van Asselt, 2018; Le Billon & Kristoffersen, 2020; Naidu et al., 2021; Rempel & Gupta, 2022). Furthermore, the goals of the Paris Agreement (UNCC, 2019) to keep a global temperature increase below 2°C and with net zero emissions will not be met before 2070 (Shears, 2019). Within this limited timeframe, immediate actions towards transitioning to a low‐carbon energy system, endorsing products with lower carbon intensity such as renewable electricity, biofuels and hydrogen are undeniably needed. Collaboration between industry and academia, as emphasized by Shears (2019), is critical for achieving the goals of the Paris Agreement.

At COP28 there were calls for greener alternatives to fossil fuels ranging from wind power, solar power (thermosolar and photovoltaic) to ocean waves and bioenergy. For the first time, a proposal to end fossil fuels use was made, however, the steps to phase out fossil fuels, outlined in COP28, remain unclear. Dramatic changes, encompassing the transport sector and the chemical industry, clear contributors to anthropogenic pollution and high energy consumption, are required (Ramos & Duque, 2019). Leading scientists at COP28 highlighted the crucial role of microbes in combating climate change, advocating for their inclusion in models aimed to ameliorate the effects of climate change. Microbes serve as catalysts in all biogenic cycles, which are endangered by human misbehaviour, and are key in preventing planetary collapse (Anonymous, 2023; Bourzac, 2023a, 2023b; Gewin, 2023).

Biofuels produced from crops have been key in promoting renewable energies; however, the ‘food versus fuel’ debate dominated in the first decade of the 21st century, and several International agencies (OECD, FAO and the EU) concluded that food commodity prices were impacted by the production of biofuels (Tenenbaum, 2008). This gave rise to policies promoting second‐generation (2G) biofuels from non‐human‐consumption feedstocks, mainly utilizing lignocellulosic residues for ethanol production (Mohr & Raman, 2013; Ramos et al., 2022; Somerville et al., 2010; Valdivia et al., 2016). Although nowadays ethanol is being used mainly as a blend with gasoline, with the current aim to suppress fossil fuels, its future may not appear so brilliant. However, it should be noted that combustion engines can use pure butanol, and therefore this biofuel produced from 2G residues could be crucial for transitioning towards net zero emissions (Re & Mazzoli, 2023).

Current biotechnological tools enable the production of a wide range of non‐fossil fuels and chemicals supported by advances in Synthetic biology (SynBio) that allow the construction of biosynthetic pathways new to nature. SynBio holds promises in the chemical and energy sectors, with potential for microbial synthesis of many organic compounds (Keasling et al., 2021; Li et al., 2023; Pfleger & Takors, 2023; Ramos et al., 2022; Ramos & Duque, 2019), and it is an appealing field for early‐career researchers in biosciences, due to its recognized economic potential (Shears, 2019). Innovative approaches in SynBio to convert solar energy into dense energy carriers represent a novel and attractive research area, because interdisciplinary collaboration between biologists, electrochemists and engineers to overcome technical and engineering challenges are faced (Fabris et al., 2020; Shears, 2019).

In the move to the new ‘green’ chemistry, based on 2G lignocellulose, technological challenges remain, including the construction of cost‐effective bioprocessing plants and efficient extraction methods of products from fermentation broths (Bele et al., 2023; de Assis et al., 2017; Ramos et al., 2017; Stephen et al., 2012). One of the key challenges discussed in 2G technology regarding profitability of the 2G processes is using the leftover lignin for further synthesis of value‐added compounds, emphasizing product recycling as required by the circular economy concept (Re & Mazzoli, 2023; Valdivia et al., 2016).

To sum up, the growing global population poses challenges not only in terms of energy and chemicals but also in increasing food production and developing energy‐efficient logistics. Addressing root ecological issues, promoting sustainable practices, transitioning to renewable energy sources and minimizing food spoilage (Sun et al., 2022), is urgent. Keeping microbes in mind should accelerate the transition steps.

## AUTHOR CONTRIBUTIONS

**Juan Luis Ramos:** Conceptualization, funding acquisition writing–review editing. **Ana Segura:** writing–review editing.

## FUNDING INFORMATION

Work in our laboratories was supported by a grant from the Agencia Estatal de Investigación grant TED2021‐1299632BI00 and a grant from the European Commission PREPSOIL, Grant Agreement No. 101070045, HORIZON CSA.

## CONFLICT OF INTEREST STATEMENT

The authors declare no conflict of interest.
